# Photodynamic Therapy Induced Cell Death Mechanisms in Breast Cancer

**DOI:** 10.3390/ijms221910506

**Published:** 2021-09-29

**Authors:** Dimakatso R. Mokoena, Blassan P. George, Heidi Abrahamse

**Affiliations:** Laser Research Centre, Faculty of Health Sciences, University of Johannesburg, P.O. Box 17011, Johannesburg 2028, South Africa; drmokoena2@gmail.com (D.R.M.); blassang@uj.ac.za (B.P.G.)

**Keywords:** apoptosis, necrosis, cytotoxicity, breast cancer, photodynamic therapy (PDT)

## Abstract

Breast cancer is the second most common cancer globally and the pioneering cause of mortality among women. It usually begins from the ducts or lobules, referred to as ductal carcinoma in situ, or lobular carcinoma in situ. Age, mutations in Breast Cancer Gene 1 or 2 (BRCA1 or BRCA2) genes, and dense breast tissue are the highest risk factors. Current treatments are associated with various side effects, relapse, and a low quality of life. Although conventional treatments, such as surgery and chemotherapy, have been used for decades, their adverse side effects on normal cells and tissues pose a major weakness, which calls for a non-invasive treatment option. Photodynamic therapy (PDT) has proven to be a promising form of cancer therapy. It is less invasive, target-specific, and with reduced cytotoxicity to normal cells and tissues. It involves the use of a photosensitizer (PS) and light at a specific wavelength to produce reactive oxygen species. One of the reasons for the target specificity is associated with the dense vascularization of cancer tissues, which tends to increase the surface area for the PS uptake. Photosensitizers are light-sensitive molecules, which result in cancer cell destruction followed by light irradiation. Depending on the localization of the PS within the cancer cell, its destruction may be via apoptosis, necrosis, or autophagy. This review focuses on the breast cancer etiopathology and PDT-induced cell death mechanisms in breast cancer cells.

## 1. Introduction

Breast cancer is a complex disease resulting from the uncontrolled growth of cells in the breast, forming a mass or sheet of cells, known as a tumor. It is the most commonly occurring cancer in women and the second most common cancer in general [1]. It is estimated that more than 90% of breast cancers are non-malignant at the time of early diagnosis; however, due to lymphatic and hematological systems, cancer tends to metastasize to other parts of the body over time [1,2]. Breast cancer is linked to several distinct life events, such as genetic mutations to the BRCA1 (breast cancer gene 1) and BRCA2 (breast cancer gene 2) genes, high breast density, family history of the disease, late full-term pregnancy, lack of physical activity, and smoking and alcohol consumption [3,4]. Another uncommon yet increasing form of breast cancer is inflammatory breast cancer, which accounts for 1% to 5% of all types of breast cancers, characterized by the warm, red, and swollen appearance of the breast [5,6].

Many variables determine the treatment options for breast cancer. These may include, but are not limited to, the type of breast cancer, its stage, and overall health and preference of the patient. For example, a patient with a localized tumor may be treated with surgery; depending on tumor progression, the initial therapy could be combined with chemotherapy or radiation therapy. If the breast cancer has spread to other areas of the body, drug-based therapies are an ideal option. These include chemotherapy, hormone therapy, targeted therapy, and immunotherapy, as these therapies can reach cancer cells in any part of the body [7,8]. The major disadvantage of the existing therapies is the debilitating side effects, which include chronic pain, failure, and relapse. Although they have been used for decades, survival rate is estimated at 5 years in 80% of cases in high-income countries and only 40% in low-income countries [9].

Photodynamic therapy (PDT) is a form of light therapy with promising therapeutic prospects for cancer treatment. It involves a photosensitizer (PS), visible light at a specific wavelength, and molecular oxygen [10,11]. It results in cancer cell destruction by inducing apoptosis, necrosis, and autophagy. The reason for its limited use is linked to the lack of detailed understanding by clinicians [12]. Thus far, PDT is observed to induce cancer cell death by the activation of various cell death pathways linked with B-cell lymphoma-2 (Bcl-2), family members, caspases, and the apoptosis-inducing factor. In the case of the unavailability of the apoptotic pathway, cell death may be induced via the necrotic or autophagic pathway, as well as by the activation of the innate immune response. The cell death induction by PDT depends on several factors, such as the localization of the photoactive agent (photosensitizer) intracellularly, cell genotype, and PDT parameters. Understanding the different pathways involved in cell death mechanisms is critical for the effectiveness of PDT [13]. This review aims to discuss the influence of PDT on breast cancer cell death mechanisms.

## 2. Breast Cancer Etiopathology

The breast consists of different tissue, ranging from fatty to dense tissue. Breast tissue spreads from the collarbone to the lower ribs, sternum (breastbone), and armpit. Each breast is comprised of 15–20 segments named lobes, and the individual lobes consist of smaller sacs, or lobules (glands). These lobules produce milk in lactating women. The lobes and lobules are joined to the nipple by tubes called ducts, which carry milk to the nipple. The nipple is located at the center of the areola, which is the dark area of skin surrounding the nipple. The breast and armpit contain lymph nodes that belong to the lymphatic system, which is a network of nodes and tubes that drain fluid (lymph) and transport white blood cells (immune cells involved in fighting against infections). The remainder of the breast consists of fatty and connective (or fibrous) tissue [1,2]. There are multiple reasons why breast cancer occurs, and most of them are associated with genetic mutations that can be hereditary or resulting from lifestyle or environmental influences on a gene or set of genes. It is understood that 5–10% of breast cancer cases are due to mutations of the breast cancer genes (BRCA1 and 2), with 25% of cases occurring in patients under the age of 30 years [3]. There are also reproductive factors that contribute, which include early onset of menorrhea before the age of 12 years, delayed childbearing and childbirth after the age of 30 years, nulliparity, and menopause after the age of 55 years [3,4]. Exposure to exogenous hormones as a contraceptive or hormone replacement for menopause is also a well-known pre-disposer to breast cancer [5].

### 2.1. Stages of Breast Cancer

Upon diagnosis, breast cancer has to be classified by looking at the tumor size, nodal status, and metastasis [6]. Staging of breast cancer is also used to describe whether the disease is in the early stages of zero to one (0–I), or in its late-stage, from two to four (II–IV) [7]. Stage zero is the non-invasive ductal carcinoma in situ (DCIS), while stages I to IV are the invasive types of breast cancer. Staging offers a mutual way of relating cancer, so doctors can work collectively in organizing the best treatments for the patients [8]. Staging is clinically performed by looking at the physical appearance of the breast and pathologically by removing the breast tissue and lymph nodes during surgery [9].

Breast cancer grade tends to differ from the stage, in that there are three grades used, which measure how different the morphology of cells compared to normal breast cells are, as well as the size and speed of the metastasis [10]. Cells in grade one look the most similar to normal breast cells and the tumor is usually slow-growing. Grade two cells look less similar to normal cells and the tumor is fast-growing. Cells in grade three look completely different compared to normal breast cells and are usually fast-growing [10,11]. This leads to variations in biomarker subtypes in cytological and histological samples. These include the human epidermal growth factor receptor-2 (HER-2), estrogen receptor (ER), progesterone receptor (PR) markers, and the triple-negative breast cancer (TNBC) markers, which further influence the tumor characteristics and prognosis. As a result, the treatment needs to be planned accordingly.

### 2.2. Breast Cancer Therapies

Surgery, chemotherapy, and radiation therapies are the main forms of cancer therapies provided for patients. Breast cancer therapy options are dependent on the severity and extent of cancer metastasis. It is said that about 90% of breast cancers are non-metastatic at the time of diagnosis. This renders the treatment option goals to be around eradicating the tumor completely and preventing its recurrence [12]. This tends to be complicated to achieve, as the molecular composition of the cancer plays a huge role in the recurrence of the cancer, as observed in TNBC cancers. There are a lot of side effects associated with breast cancer therapies. Patients often suffer from reduced quality of life upon treatment, due to the adverse effects that come with the treatment. These would normally include, nausea and vomiting due to chemotherapy, impaired physical functioning associated with surgery, and radiation therapy. In addition, there may also be pain, impaired sleeping patterns and peripheral neuropathy associated with chemotherapy, fatigue, depression, and anxiety, just to mention a few of the side effects [13].

Surgery is a preferred form of treatment, depending on the size of the tumor and whether or not it has metastasized to other organs [14]. It usually involves a lumpectomy, also known as breast conservation therapy, with the partial removal of the breast, as well as mastectomy, which entails the complete removal of the breast tissue. Without a doubt, surgery is invasive and usually leaves the patient with undesirable effects, such as inflammation, sclerosis, tenderness, distorted appearance of the breast, asexuality, depression, and loss of self-image [15,16]. Radiation therapy is usually used in combination with surgery, for the complete removal of tumor cells [14].

## 3. Photodynamic Therapy (PDT)

Photodynamic therapy (PDT) is a unique method of eradicating a variety of diseases that necessitate the removal of pathological cells. It has received much attention over the years due to its specificity, minimal to non-invasive nature, and selective cytotoxicity towards malignant cells, which means that normal cells are preserved during treatment as compared to conventional therapies [17]. PDT also preserves the local cytoarchitecture/extracellular matrix (ECM). This improves tissue repair and cosmesis. PDT entails the use of visible light, a photoactive agent known as the photosensitizer (PS), and molecular oxygen to eradicate malignant cells and pathogenic bacteria. The PS is disseminated directly on the tumor site, either intravenously, depending on the pathology of the tissue [18], or orally, to achieve optimum tissue PS concentration [19,20]. PS dissemination and localization is then followed by the irradiation of light at a specific wavelength, leading to the excitation of the PS from its ground state (singlet state) to its excited singlet state. With continued irradiation, the PS undergoes an intersystem crossing from its excited singlet state to its excited triplet state. This may lead to type I reactions, in which the PS in its excited triplet state reacts with biomolecules, resulting in the production of reactive oxygen species (ROS). This leads to oxidative damage of the intracellular elements within the targeted cells or tissue [21]. Alternatively, type II reactions in which the PS reacts with molecular oxygen to produce excited singlet oxygen species [22], can occur. PDT results in vessel constriction, blood flow inhibition, and blood vessel collapse when the target is via the tumor vasculature. This inhibits the nutrient supply to the tumor, thus leading to tumor cell destruction. Moreover, depending on the type of PS used, the innate immune system becomes activated to further aid in tumor cell demolition. Therefore, choosing an appropriate PS is vital for the effectiveness of PDT [18,23].

**Figure 1:**
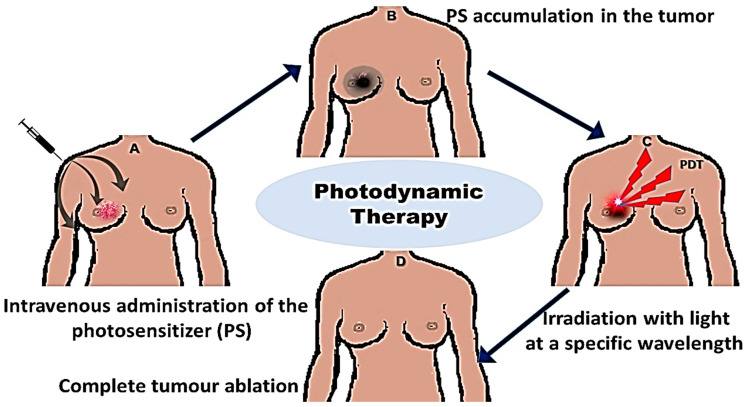
PDT treatment scheme. Intravenous administration of the PS, which localizes on the tumor site and results in tumor ablation, when light at a specific wavelength is applied.

PDT is mostly known to induce cell death via apoptosis, however, during numerous experiments over the years, it has been observed that PDT induces cell death via necrosis, or autophagy, as well as in the aforementioned mechanisms, depending on the localization of the PS within the tumor cells. The PS can localize several organelles within the cell, which include the plasma membrane, endoplasmic reticulum (ER), mitochondria, Golgi apparatus, cytoplasm, and lysosomes. An acute stress response occurs on cells after PDT, thus leading to the change in calcium and lipid metabolism, resulting in the production of stress response mediators and cytokines [24]. This results in the activation of protein kinases and the expression of transcription factors. Cellular responses centered from the mitochondrion are known to result in apoptosis, which involves cytochrome c release and caspase activation. They also include the damage of specific proteins, such as the Bcl-2, which further augment apoptosis. PDT induces cell death in a variety of pathways; the increased PDT-induced oxidation results in a build-up of oxidized proteins, which leads to an ER-stress response and therefore, an increased proteasome inhibition. This leads to an increased NOXA level and the activation of caspase 9, which ends in apoptosis [25]. Figure 1 shows the PDT treatment scheme from the PS intravenous administration, selective accumulation in the tumor, PDT light irradiation, and tumor demolition.

Although PDT has been known for decades, its acceptance as an alternative therapy is very limited, due to factors such as light delivery in deeply located tumors [26] and PS selection. The basis of PDT treatment is using a PS that becomes activated at wavelengths within the therapeutic window, which is within the blue- and red-light regions. However, compared to other conventional therapies, PDT, as a non-conventional therapy, has demonstrated the ability to reduce long-term morbidity, with a favorable decrease in recurrence and metastasis, which remains the main cause of failure in conventional therapies, such as chemo- and radiation therapy [23]. Similarly to any other therapy, PDT has its own challenges, ranging between choosing the appropriate PS with the best tumor cell selectivity and increased cellular uptake, to increasing the PS efficacy using antibody conjugated PS and nano carriers. As a result, many studies are devoted to solving this challenge, by introducing drug delivery mechanisms using nanoparticles. Some studies have demonstrated that PDT alone might not fully achieve the desired results; however, in combination with other therapies, such as chemotherapy, surgery, and photothermal therapy (PTT), complete tumor demolition is achievable. Photothermal therapy entails the use of nanoparticles or photosensitizers to induce cancer cell death by irradiation at electromagnetic radiation with preferably infrared wavelengths. This is an extension of the PDT approach and occurs when the PS or nanoparticle inside the tumor releases heat following irradiation with light at a specific wavelength [27]. Examples are given thereafter:

Xu and other colleagues showed that the combination of targeted PDT and photothermal therapy (PTT) has the potential of effectively treating HER2 positive breast cancer. They observed an increased cellular uptake of 5-aminolevulinic acid PS conjugated to functionalized gold-nanorods, Cy7.5 (Cyanine 7.5) fluorescent dye, anti-HER2, and anti-CD44 (cluster of differentiation-44) antibody. They observed a significantly increased level of ROS and heat production in MCF-7 breast cancer cells, as compared to individual therapies. They demonstrated that HER2 and CD44 receptors facilitated a two-fold targeting, which significantly enhanced cellular uptake of their conjugate. This finding has led to the conclusion that the combination of PDT and PTT has superior antitumor effects in both in vitro and in vivo models [28]. Riley and colleagues also observed a synergistic induction of cell death in MDA-MB-231 TNBC cells, using the combination of PDT and PTT. They observed that reduced doses of light induced apoptotic cell death [27].

Gabrielle and other colleagues used the Hypericin (HYP) photosensitizer loaded with pluronic^®^123 (P123) micelles on MCF-7 breast cancer cells and observed increased cell-binding capacity and selective cellular uptake in MCF-7 cells, in comparison to the normal breast cells (MCF-10A). They also observed that the HYP/P123 combination, localized in the mitochondrion and endoplasmic reticulum, resulted in PDT cell death by necrosis. Their study lead to the conclusion that the HYP/P123 combination has the potential of increased specificity and effectiveness for breast cancer treatment via PDT in vivo preclinical evaluations, due to the inhibition of tumor cell migration [29].

There are several in vivo studies reported on the effectiveness of PDT in eradicating cancer. Using an in vivo mouse model injected with 4TI metastatic cancer cells on the left oxter region, Wang and other colleagues observed an increased survival rate of mice following the PDT treatment, and they also observed an inhibition of tumor progression and metastasis [30]. Hoi and other colleagues observed a substantial inhibition of breast tumor growth in in vivo mouse xenographs with MCF-7 cells by PDT [31]. Duanmu and other colleagues observed a safe and effective destruction of multidrug resistant MCF-7 cells in chemoresistant breast cancer-injected mouse models in vitro, using targeted PDT of the tumor vasculature and breast cancer cells [32].

### Clinical Applications of PDT for Cancer Treatment

PDT is currently used for dermatological applications. It is used for the treatment of actinic keratoses and early non-melanoma skin cancers [33]. It is mainly applied in cutaneous neoplasms, such as in the chest wall of breast cancer patients following surgery, head and neck tumors [34], and early stages of oral cavity and oropharynx neoplasms, etc. [35]. It is used in an attempt to prevent cutaneous recurrence following mastectomy, radiation, and chemotherapy [36]. PDT has received approvals from the Food and Drug Administration (FDA) and the European Medicine Agency as a palliative therapy and therapeutic treatment for solid tumors and pre-cancerous lesions [37].

Morrison and other colleagues showed the effectiveness of PDT in the treatment of cutaneous and subcutaneous metastatic breast cancer, following mastectomies. They observed a positive histologic response, indicating tumor apoptosis and regression. They also reported an improved quality of life in the patients [38]. Table 1 shows the summary of clinical trials dedicated to PDT on breast cancer, the phases of the study, and the stages of the trials.

There are not a lot of clinical data showing the effectiveness of PDT in breast cancer studies. However, the available in vitro and in vivo studies suggest that PDT may be an effective treatment modality for superficial cancers with 2–10 nm depths [33].

## 4. Cell Death Mechanisms

Biologically, cell death in normal and pathological conditions follows one of the many different types of cell death mechanisms. Depending on the stimulus, the cell death can be complex and regulated, or simple and unregulated. For the longest time, cell death was described by the morphological appearance of the dying cell, and whether or not it followed a more physiologic and regulated process. This resulted in three commonly used terms: Apoptosis, necrosis, and autophagy [47,48,49]. Due to this vagueness in the classification of cell death mechanism, the Nomenclature Committee on Cell Death (NCCD), proposed a set of guidelines that include morphological, biochemical, and functional perspectives of the process to define and classify different types of cell death mechanisms (Table 2) [50].

## 5. Cell Death Mechanisms in PDT Treated Breast Cancer Cells

**Figure 2:**
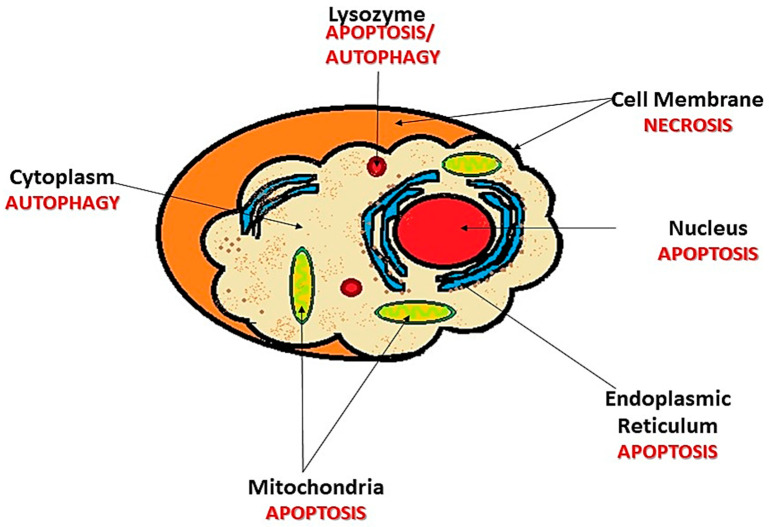
Photosensitizer (PS) localization in relation to the cell death pathway, activated during PDT. PS can localize in the cytoplasm and cause autophagy. Lysosomes undergo apoptosis and/or autophagy, cell membrane undergoes necrosis, and nucleus, endoplasmic reticulum, and mitochondria undergo apoptosis.

Literature shows that there is no single pathway leading to cell death after PDT treatment [24,62]. This partly explains why PDT succeeds in eradicating breast cancer cells even when they display varying invasive properties [63]. A lack of effective treatments for aggressive breast cancer is still a major global health problem. Breast cancer is a very complex disease, presenting with different levels of aggressive properties, which render it difficult to treat using conventional therapies. Moreover, other studies on breast cancer do not seem to have a clear explanation of how PDT eradicates tumor cells. However, PDT has been observed to induce cell death via direct cell damage or cytotoxicity, vasculature shutdown, and activation of the immune response, depending on where the PS localizes within the cell [24,62] (Figure 2). Direct cell damage is associated with apoptosis in cases where the PS localizes in the mitochondria and results in cytochrome c release and Bcl-2 damage. Apoptosis is also observed when the PS localizes in the cytoplasm, in which damage to the nuclear factor kappa B (NFĸB) pathway occurs [64]. This damage to the NFĸB pathway is important, as it hinders its activity of anti-apoptotic gene stimulation [24]. Autophagy takes place when the PS localizes in the endoplasmic reticulum (ER), leading to the Beclin-1 and the mechanistic target of rapamycin (mTOR) protein activation. Furthermore, necrosis is evident when there is cell membrane disintegration caused by the PS [62,65]. The destruction of the tumor vasculature (indirect cytotoxicity) is characterized by microvasculature stasis, which results in hypoxia and the local depletion of nutrients, resulting in tumor regression [66].

### 5.1. Apoptosis

**Figure 3:**
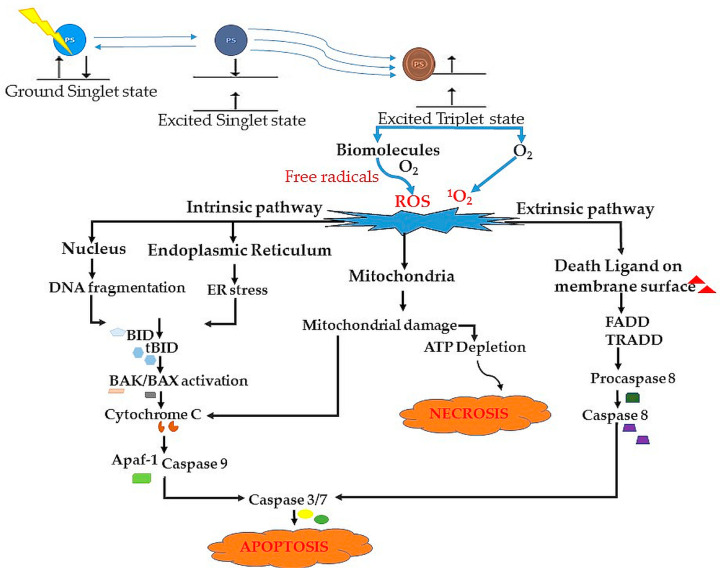
PDT activation of the apoptotic and necrotic pathways. During PDT, the PS becomes activated from its singlet ground state to its excited singlet state. With continued light irradiation the PS undergoes an intersystem, crossing to its excited triplet state. Two types of reactions can occur depending on the availability of free radicals and molecular oxygen. These include type I and type II reactions. In type I, photo-oxidation occurs with the use of free radicals, while in type II reactions it occurs with the use of molecular oxygen. The apoptotic pathway will take place via the intrinsic or extrinsic pathway. Intrinsic pathway results from DNA fragmentation and ER stress. Extrinsic pathway is activated by the death ligand on the membrane surface. Necrosis will occur due to ATP depletion.

Apoptosis is an innate process that cells experience in response to a trigger. The word apoptosis is a description of a specific type of cell death, coupled with several cellular morphological changes, such as a reduction of cellular volume, chromatin reduction, rounding up of the cell, nuclear condensation, and plasma membrane blebbing, to mention a few [67,68]. Apoptosis requires consecutive activation of a set of enzymes, such as the cysteine aspartate-specific proteases, known as caspases and endonucleases [59]. It continues to be classified as resulting from intrinsic and extrinsic factors. Intrinsic apoptosis is a form of regulated cell death resulting from a variety of microenvironment disturbances, such as deoxyribonucleic acid (DNA) damage, reactive oxygen species (ROS), endoplasmic reticulum stress, replication stress, and growth factor withdrawal [24]. It is a non-receptor mediated pathway, facilitated by the cleavage of Bid (BH3 interacting domain death agonist), a Bcl-2 homology 3 (BH3)-only protein. Consecutively, Bid translocate to the mitochondria to activate Bax and Bak proteins (Figure 3). This results in mitochondrial permeability leading to the release of cytochrome c, thus the oligomerization of Apaf-1 (apoptotic peptide activating factor 1). This is followed by the recruitment of procaspase 9, the activation of caspase 9, through the CAR-CARD interaction and thus the activation of caspase 3/7, resulting in programmed cell death [69,70]. The extrinsic pathway is a transmembrane receptor-mediated pathway, involving the activation of FADD and therefore the recruitment of pro-caspase 8. This leads to the activation of caspase 8, followed by automatic activation of caspase 3, resulting in apoptosis [60,70]. As mentioned above, the stimulation of apoptosis following PDT is highly dependent on the intracellular localization of the PS. For instance, when the PS localizes in the lysosome, the irradiation will result in the destruction of the lysosomal membrane, thus leading to the release of cathepsins and proteases into the cytosol. Furthermore, this leads to the cleavage of Bid, a proapoptotic protein, into the truncated Bid (tBid) [71].

### 5.2. Necrosis

Necrosis is another type of cell death, resulting from cell membrane integrity loss due to severe cell injury [59] (Figure 3). The consequences of necrosis include the release of cellular contents into the surrounding tissues and therefore, triggering an inflammatory response [72]. Moreover, necrotic cells tend to go through a process of oncosis, which ends in cell rupture. In contrast to apoptosis, necrosis is an energy-independent form of cell death, where the cells are damaged severely by a sudden shock (radiation, heat, chemicals, hypoxia, etc.) For decades, necrosis has always been accepted as the only mode of cell death post-PDT treatment. This seemed true when observed from the endpoint state of cells in vivo. This is because the presence of cellular fragments, resulting from the late apoptotic cells, was assumed to have been due to necrotic cell death.

In recent years, due to a wider understanding, cell death mechanisms have become more comprehensive. Moreover, the modalities are not only limited to apoptosis, necrosis, and autophagy, but to a whole list of terms, which have been incorporated in the pursuit of a proper description of cell death. Despite the complexity of the novel understanding of the details of physiological and molecular cascades concerning cell death pathways, the opportunity for improving PDT efficacy through combination therapies has been granted.

### 5.3. Autophagy

Autophagy is normally a cell recycling process, in which a cell undergoing destruction forms vacuoles called autophagosomes, which encapsulate organelles and the cytosol. However, in certain instances, it aids in enhancing cell death in apoptosis-lacking cells [51,73]. In PDT, autophagy can result in cell survival or death, depending on the intensity of the cytotoxicity produced during treatment. This is achieved by the recycling of the damaged mitochondria or the ER before apoptosis can be stimulated. However, in cases where PDT cytotoxicity is optimal, both the apoptotic and the autophagic pathways will result in cell death. Furthermore, autophagy is observed in apoptosis-resistant cells [74].

## 6. Indirect Cytotoxicity via Destruction of the Tumor Vasculature

**Figure 4:**
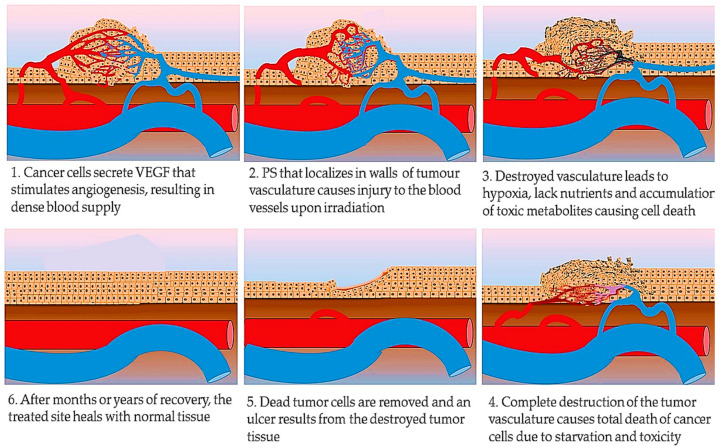
Tumor vasculature targeted PDT. Depletion of the tumor vasculature results in tumor nutrient deprivation leading to complete tumor tissue destruction.

One of the features of a tumor that enables it to survive in the body is the increased vascularization that occurs in tumor tissues. This results from the increased secretion of VEGF by tumor cells that initiate neovascularization in the tissue. This is a necessary mechanism of tumors to increase perfusion of blood into the expanding tissue, that supply the cancer cells with enough oxygen, nutrients, and enable the excretion of metabolic by-products, otherwise, the tumor would die. PDT takes advantage of this occurrence. After PS administration, some PS may localize in the walls of the tumor vasculature. Upon irradiation, the PS absorbed by the endothelial cells of the tumor vasculature undergoes a similar activation process. This destroys the blood vessel walls and leads to the disruption of blood supply to the tumor tissue. When the blood supply is disrupted, the dense tumor cells are starved of nutrients, oxygen, and other blood elements. Additionally, the metabolic waste products from the cell metabolism start accumulating in the tissues [75,76]. All these together lead to cell death, as summarized in Figure 4.

This is not the case in conventional cancer therapies, in which tumor cells have been observed to remake their metabolism for survival and proliferation under hypoxic milieu. The tumor micro-environment changes the chemokines, cytokines, and juxtacrine cell interactions, stimulating the surrounding normal cells, such as fibroblast and endothelial cells, for survival sustenance. This leads to the differentiation of fibroblasts into cancer-associated fibroblasts, thus producing ECM for the tumor cells. This leads to treatment resistance and tumor recurrence [77,78]. Anticancer drugs cause toxicity through different mechanisms that have been substantially reviewed by many researchers [15,79,80,81].

## 7. Post-PDT Activation of the Immune System

Another important feature of tumors that enables their survival in the body is the evasion of immune recognition by cells of the immune system. When cancer develops, pathological antigens that should be potentially recognized by the immune system, are obscured from immune action. This occurs by different mechanisms, including the presentation of surface molecules that stop immune cells from causing an immune attack, and the secretion of molecules that suppress the action of the immune cells, along with many other mechanisms [82]. Although primarily, PDT is thought to cause direct cytotoxicity, the effects of PDT directly activate the immune system.

The dying cells and leakage of cellular debris result in the release of cytokines and tumor necrosis factors (TNFs) that enhance the inflammation and initiate a cascade of other immune response mechanisms [83,84]. Other chemical mediators of inflammation and immune reaction, including leukotrienes, acute phase proteins, complement system factors, histamine, granulocyte colony-stimulating factors (G-CSF), prostaglandins, and many others are also released post-PDT cytotoxicity [84,85]. Furthermore, dying cells in the body, including tumor cells upon treatment, always release molecules such as alarming and damage-associated molecular patterns (DAMPs), into the vascular system, which in turn, alerts the human body about a potential threat at the injured site [86,87]. These molecules are secreted outwardly into the blood to elicit the innate immune system. At this point, the cancer escaping method by the cancer cells is compromised and the innate immune system cells, such as macrophages, dendritic cells (DCs), natural killer (NK) cells, lymphocytes, and particularly neutrophils, are activated. Thus, PDT results in not only inflammation, but also immunogenic cell death of the cancer cells, with a subsequent clearance by the immune system.

In addition to the innate immune system activation, post-PDT innate immune responses initiate the stimulation of the adaptive immune reaction. Antigen-presenting cells (APCs) work to produce cells of the adaptive immune system, including helper T cells, cytotoxic T cells, and regulatory T lymphocytes. This occurs in a process that activates the adaptive immune system to complement the action of the innate immune system [24]. Interestingly, activation of the adaptive immune system results in the development of a permanent adaptive immunity against memory T cells, serving a long-lasting antitumor immunity that controls tumor metastasis and helps to avoid the recurrence of cancer post-PDT [59].

## 8. Conclusions

Breast cancer is a complex disease resulting from the uncontrolled growth of cells in the breast. Its complexity calls for a better treatment modality with fewer side effects, while still remaining effective at treating all types and stages of breast cancer. As in all types of cancers, late diagnosis, followed by cancer drug resistance, is one of the most challenging factors when it comes to treatment options. Different types of breast cancers have different treatment responses, which should be understood. Photodynamic therapy (PDT) is known to prompt cell death, depending on the subcellular localization of the photosensitizers (PS) and the degree of inflammation, using the four main cell death pathways: Apoptosis, necrosis, autophagy, and the immunogenic pathway. However, this does not rule out other cell death pathways. Even so, PDT has the potential to initiate different cell death pathways concurrently, following damage to the lysosomes and mitochondria. This increases the potential of consecutive subcellular organelle targeting, thus increasing the efficacy of PDT. In conclusion, the ability of PDT to stimulate the innate immune system renders it a promising form of treatment over other therapies. However, factors such as early diagnosis of the tumor, antigen classification of each type of breast cancer, and understanding of resistance patterns should also be put under consideration. Further studies need to be conducted in order to understand the tumor resistant patterns, possible antigen classifications and targets for each type of breast cancer, activation and suppression of the immune system, and dose-specific approaches to fine-tune the limited application of PDT.

## Figures and Tables

**Table 1:** Clinical trials on PDT of breast cancer and related conditions.

Clinical Trial Number	Study Details	Stage	Country	Phase
NCT02939274	Confirmed stage IIIb and IV breast cancer treatment with continuous low-irradiance PDT using Verteporfin.	Unknown	USA	II [39]
NCT00862901	PDT study on patients with chest wall progression of breast cancer.	Completed	USA	I [40]
NCT02872064	PDT treatment of primary breast cancer diagnosed patients and patients that received mastectomy or local wide excisions of the breast.	Completed	UK	I/IIA [41]
NCT01262716	PDT for the treatment of chest wall progression of breast cancer.	Completed	USA	N/A [42]
NCT00023790	PDT treatment of skin cancer or solid tumors metastatic to the skin.	Terminated	Ireland and USA	I [43]
NCT03833726	PDT for the treatment of breast cancer associated treatment side effects.	Unknown	Brazil	N/A [44]
NCT00028405	Refractory solid tumor treatment with PDT.	Completed	USA	I [45]
NCT03713203	Use of a PDT device to treat extra-mammary Paget’s disease of the vulva.	Recruiting	France	N/A [46]

Information taken from: clinicaltrials.gov, accessed on 10 August 2021.

**Table 2:** Cell death mechanisms according to the Nomenclature Committee on Cell Death.

Mechanism	Mode of Action	References
Apoptosis	Occurs due to physiological events.	[51]
Necrosis	Inflammatory type of cell death.	[52]
Necroptosis	Combination of apoptosis and necrosis mode of actions.	[53]
Autophagy	Stimulated by dysfunctional organelles.	[54]
Pyroptosis	Dependent on plasma membrane pore formation by the gasdermin protein family.	[55]
Ferroptosis	Oxidative disturbances of the intracellular microenvironment controlled by GPX4.	[56]
Parthanatos	Initiated by PARP1 activation, AIF, and MIF dependent DNA degradation.	[57,58]
LDCD	Primary LMP and cathepsins and slight involvement of MOMP and caspases.	[56]
ADCD	Autophagic machinery.	[50]
ICD	Activation of the immune response in immunocompetent hosts.	[59]
Intrinsic apoptosis	Disturbances in the extracellular space distinguished by caspase 9 and caspase 3.	[60]
Extrinsic apoptosis	Disturbances caused by the extracellular microenvironment. Propagated by caspase 8 and 3.	[60]
MPT-driven necrosis	Intracellular microenvironment disturbances.	[61]

ADCD: Autophagy-dependent cell death, AIF: Autophagy-dependent cell death, DNA: Deoxyribonucleic acid, GPX4: Glutathione peroxidase 4, ICD: Immunogenic cell death, LMP: Lysosomal membrane permeabilization, LDCD: Lysosome-dependent cell death, MIF: Macrophage migration inhibitory factor, MPT: Mitochondrial permeability transition, MOMP: Mitochondrial outer membrane permeability, PARP1: Poly (ADP-ribose) polymerase 1, ROS: Reactive oxygen species.

## Data Availability

Not Applicable.

## References

[1] Alkabban F.M., Ferguson T. (2020). Breast cancer. StatPearls.

[2] Mahvi D.A., Liu R., Grinstaff M.W., Colson Y.L., Raut C.P. (2018). Local Cancer Recurrence: The Realities, Challenges, and Opportunities for New Therapies. CA Cancer J. Clin..

[3] Doren A., Vecchiola A., Aguirre B., Villaseca P. (2018). Gynecological-Endocrinological Aspects in Women Carriers of BRCA1/2 Gene Mutations. Climacteric.

[4] Narod S.A. (2018). Personalised Medicine and Population Health: Breast and Ovarian Cancer. Hum. Genet..

[5] Chen W.Y. (2008). Exogenous and Endogenous Hormones and Breast Cancer. Best Pract. Res. Clin. Endocrinol. Metab..

[6] Giuliano A.E., Edge S.B., Hortobagyi G.N. (2018). Eighth Edition of the AJCC Cancer Staging Manual: Breast Cancer. Ann. Surg. Oncol..

[7] Warner E.T., Tamimi R.M., Hughes M.E., Ottesen R.A., Wong Y.-N., Edge S.B., Theriault R.L., Blayney D.W., Niland J.C., Winer E.P. (2012). Time to Diagnosis and Breast Cancer Stage by Race/Ethnicity. Breast Cancer Res. Treat..

[8] Feng X., Zhang R., Liu M., Liu Q., Li F., Yan Z., Zhou F. (2018). An Accurate Regression of Developmental Stages for Breast Cancer Based on Transcriptomic Biomarkers. Biomark. Med..

[9] Elyasinia F., Keramati M.R., Ahmadi F., Rezaei S., Ashouri M., Parsaei R., Yaghoubi M., Elyasinia F., Aboutorabi A., Kaviani A. (2017). Neutrophil-Lymphocyte Ratio in Different Stages of Breast Cancer. Acta Med. Iranica.

[10] Oluogun W.A., Adedokun K.A., Oyenike M.A., Adeyeba O.A. (2019). Histological Classification, Grading, Staging, and Prognostic Indexing of Female Breast Cancer in an African Population: A 10-Year Retrospective Study. Int. J. Health Sci..

[11] Zhang R., Chen H., Wei B., Zhang H., Pang Z., Zhu H., Zhang Z., Fu J., Bu H. (2010). Reproducibility of the Nottingham Modification of the Scarff-Bloom-Richardson Histological Grading System and the Complementary Value of Ki-67 to This System. Chin. Med. J..

[12] Waks A.G., Winer E.P. (2019). Breast Cancer Treatment: A Review. JAMA.

[13] Greenlee H., DuPont-Reyes M.J., Balneaves L.G., Carlson L.E., Cohen M.R., Deng G., Johnson J.A., Mumber M., Seely D., Zick S.M. (2017). Clinical Practice Guidelines on the Evidence-Based Use of Integrative Therapies during and after Breast Cancer Treatment. CA Cancer J. Clin..

[14] Akram M., Iqbal M., Daniyal M., Khan A.U. (2017). Awareness and Current Knowledge of Breast Cancer. Biol. Res..

[15] Vaz-Luis I., Hughes M.E., Cronin A., Rugo H.S., Edge S.B., Moy B., Theriault R.L., Hassett M.J., Winer E.P., Lin N.U. (2016). Trends in the Use of Mastectomy in Women with Small Node-Negative Breast Cancer Treated at US Academic Centers. Breast Cancer Res. Treat..

[16] Keskin G., Gumus A.B. (2011). Turkish Hysterectomy and Mastectomy Patients—Depression, Body Image, Sexual Problems and Spouse Relationships. Asian Pac. J. Cancer Prev..

[17] Agostinis P., Berg K., Cengel K.A., Foster T.H., Girotti A.W., Gollnick S.O., Hahn S.M., Hamblin M.R., Juzeniene A., Kessel D. (2011). Photodynamic Therapy of Cancer: An Update. CA Cancer J. Clin..

[18] Kwiatkowski S., Knap B., Przystupski D., Saczko J., Kędzierska E., Knap-Czop K., Kotlińska J., Michel O., Kotowski K., Kulbacka J. (2018). Photodynamic Therapy—Mechanisms, Photosensitizers and Combinations. Biomed. Pharmacother..

[19] Gossner L., Sroka R., Hahn E.G., Ell C. (1995). Photodynamic Therapy: Successful Destruction of Gastrointestinal Cancer after Oral Administration of Aminolevulinic Acid. Gastrointest. Endosc..

[20] Anand S., Yasinchak A., Bullock T., Govande M., Maytin E.V. (2019). A Non-Toxic Approach for Treatment of Breast Cancer and Its Metastases: Capecitabine Enhanced Photodynamic Therapy in a Murine Breast Tumor Model. J. Cancer Metastasis Treat..

[21] Abrahamse H., Hamblin M.R. (2016). New Photosensitizers for Photodynamic Therapy. Biochem. J..

[22] Robertson C.A., Evans D.H., Abrahamse H. (2009). Photodynamic Therapy (PDT): A Short Review on Cellular Mechanisms and Cancer Research Applications for PDT. J. Photochem. Photobiol. B Biol..

[23] Dos Santos A.F., de Almeida D.R.Q., Terra L.F., Baptista M.S., Labriola L. (2019). Photodynamic Therapy in Cancer Treatment—An Update Review. J. Cancer Metastasis Treat..

[24] Mroz P., Yaroslavsky A., Kharkwal G.B., Hamblin M.R. (2011). Cell Death Pathways in Photodynamic Therapy of Cancer. Cancers.

[25] Nunes A.T., Annunziata C.M. (2017). Proteasome Inhibitors: Structure and Function. Semin. Oncol..

[26] Ostańska E., Aebisher D., Bartusik-Aebisher D. (2021). The Potential of Photodynamic Therapy in Current Breast Cancer Treatment Methodologies. Biomed. Pharmacother..

[27] Riley R.S., O’Sullivan R.K., Potocny A.M., Rosenthal J., Day E.S. (2018). Evaluating Nanoshells and a Potent Biladiene Photosensitizer for Dual Photothermal and Photodynamic Therapy of Triple Negative Breast Cancer Cells. Nanomaterials.

[28] Xu W., Qian J., Hou G., Wang Y., Wang J., Sun T., Ji L., Suo A., Yao Y. (2019). A Dual-Targeted Hyaluronic Acid-Gold Nanorod Platform with Triple-Stimuli Responsiveness for Photodynamic/Photothermal Therapy of Breast Cancer. Acta Biomater..

[29] Gabrielle M.Z.F.D., Raquel P.S., Maiara C.M., Edilson D., Renato S.G., Gabriel B.C., Elza K., Wilker C., Noboru H., Marcia E.L.C. (2020). Selective Photodynamic Effects on Breast Cancer Cells Provided by P123 Pluronic^®^-Based Nanoparticles Modulating Hypericin Delivery. Anti-Cancer Agents Med. Chem..

[30] Wang X., Hu J., Wang P., Zhang S., Liu Y., Xiong W., Liu Q. (2015). Analysis of the In Vivo and In Vitro Effects of Photodynamic Therapy on Breast Cancer by Using a Sensitizer, Sinoporphyrin Sodium. Theranostics.

[31] Hoi S.W.-H., Wong H.M., Chan J.Y.-W., Yue G.G.L., Tse G.M.-K., Law B.K.-B., Fong W.P., Fung K.P. (2012). Photodynamic Therapy of Pheophorbide a Inhibits the Proliferation of Human Breast Tumour via Both Caspase-Dependent and -Independent Apoptotic Pathways in In Vitro and In Vivo Models. Phytother. Res..

[32] Duanmu J., Cheng J., Xu J., Booth C.J., Hu Z. (2011). Effective Treatment of Chemoresistant Breast Cancer in Vitro and in Vivo by a Factor VII-Targeted Photodynamic Therapy. Br. J. Cancer.

[33] Yanovsky R.L., Bartenstein D.W., Rogers G.S., Isakoff S.J., Chen S.T. (2019). Photodynamic Therapy for Solid Tumors: A Review of the Literature. Photodermatol. Photoimmunol. Photomed..

[34] Civantos F.J., Karakullukcu B., Biel M., Silver C.E., Rinaldo A., Saba N.F., Takes R.P., Vander Poorten V., Ferlito A. (2018). A Review of Photodynamic Therapy for Neoplasms of the Head and Neck. Adv. Ther..

[35] Karakullukcu B., van Oudenaarde K., Copper M.P., Klop W.M.C., van Veen R., Wildeman M., Bing Tan I. (2011). Photodynamic Therapy of Early Stage Oral Cavity and Oropharynx Neoplasms: An Outcome Analysis of 170 Patients. Eur. Arch. Otorhinolaryngol..

[36] Schuh M., Nseyo U.O., Potter W.R., Dao T.L., Dougherty T.J. (1987). Photodynamic Therapy for Palliation of Locally Recurrent Breast Carcinoma. J. Clin. Oncol..

[37] Falk-Mahapatra R., Gollnick S.O. (2020). Photodynamic Therapy and Immunity: An Update. Photochem. Photobiol..

[38] Morrison S.A., Hill S.L., Rogers G.S., Graham R.A. (2014). Efficacy and Safety of Continuous Low-Irradiance Photodynamic Therapy in the Treatment of Chest Wall Progression of Breast Cancer. J. Surg. Res..

[39] Rogers Sciences Inc. (2019). An Open Label, Phase II Trial of Continuous Low-Irradiance Photodynamic Therapy (CLIPT) Using Verteporfin (Visudyne®) for the Treatment of Cutaneous Metastases of Breast Cancer Cancer.

[40] Tufts Medical Center (2011). A Phase I Trial of Continuous Low-Irradiance Photodynamic Therapy (CLIPT) for Patients Failing Radiation Therapy.

[41] University College London (2018). A Phase I/IIa, Open Label, Single Site Light Dose Escalation Trial of Single Dose Verteporfin Photodynamic Therapy (PDT) in Primary Breast Cancer.

[42] Rogers Sciences Inc. (2011). A Novel Therapy for the Treatment of Chest Wall Progression of Breast Cancer.

[43] Case Comprehensive Cancer Center (2011). Phase I Trial of PC 4-PDT (NSC 676418) for Cutaneous Malignancies.

[44] Centro de Atenção ao Assoalho Pélvico (2019). Light Emitting Diode for the Treatment of Genitourinary Syndrome of Menopause Associated With Hormonal Therapy for Treating Breast Cancer: Randomized Controlled Clinical Trial.

[45] Light Sciences LLC (2005). A Multicenter Phase I Safety and Tolerability Study of the Oncolux System for Intratumoral Delivery of Non-Coherent Light for the Photoactivation of LS 11 in Patients with Refractory Solid Tumors.

[46] University Hospital (2020). An Interventional, Phase II, Non Randomized, Mono-Centric Study on the Clinical Efficacy and Safety of the Medical Device PAGETEX® as a Photodynamic Therapy Device in the Treatment of Extra-Mammary Paget’s Disease of the Vulva (EMPV).

[47] Plaetzer K., Kiesslich T., Verwanger T., Krammer B. (2003). The Modes of Cell Death Induced by PDT: An Overview. Med. Laser Appl..

[48] Notte A., Leclere L., Michiels C. (2011). Autophagy as a Mediator of Chemotherapy-Induced Cell Death in Cancer. Biochem. Pharmacol..

[49] Eriksson D., Stigbrand T. (2010). Radiation-Induced Cell Death Mechanisms. Tumor Biol..

[50] Galluzzi L., Vitale I., Aaronson S.A., Abrams J.M., Adam D., Agostinis P., Alnemri E.S., Altucci L., Amelio I., Andrews D.W. (2018). Molecular Mechanisms of Cell Death: Recommendations of the Nomenclature Committee on Cell Death 2018. Cell Death Differ..

[51] Kessel D., Reiners J.J. (2007). Apoptosis and Autophagy After Mitochondrial or Endoplasmic Reticulum Photodamage. Photochem. Photobiol..

[52] Adigun R., Basit H., Murray J. (2020). Cell liquefactive necrosis. StatPearls.

[53] Dhuriya Y.K., Sharma D. (2018). Necroptosis: A Regulated Inflammatory Mode of Cell Death. J. Neuroinflamm..

[54] Glick D., Barth S., Macleod K.F. (2010). Autophagy: Cellular and Molecular Mechanisms. J. Pathol..

[55] Wang Y.-Y., Liu X.-L., Zhao R. (2019). Induction of Pyroptosis and Its Implications in Cancer Management. Front. Oncol..

[56] Dos Santos A.F., Inague A., Arini G.S., Terra L.F., Wailemann R.A.M., Pimentel A.C., Yoshinaga M.Y., Silva R.R., Severino D., de Almeida D.R.Q. (2020). Distinct Photo-Oxidation-Induced Cell Death Pathways Lead to Selective Killing of Human Breast Cancer Cells. Cell Death Dis..

[57] Fatokun A.A., Dawson V.L., Dawson T.M. (2014). Parthanatos: Mitochondrial-Linked Mechanisms and Therapeutic Opportunities. Br. J. Pharmacol..

[58] Soriano J., Mora-Espí I., Alea-Reyes M.E., Pérez-García L., Barrios L., Ibáñez E., Nogués C. (2017). Cell Death Mechanisms in Tumoral and Non-Tumoral Human Cell Lines Triggered by Photodynamic Treatments: Apoptosis, Necrosis and Parthanatos. Sci. Rep..

[59] Nath S., Obaid G., Hasan T. (2019). The Course of Immune Stimulation by Photodynamic Therapy: Bridging Fundamentals of Photochemically-Induced Immunogenic Cell Death to the Enrichment of T Cell Repertoire. Photochem. Photobiol..

[60] Elmore S. (2007). Apoptosis: A Review of Programmed Cell Death. Toxicol. Pathol..

[61] Galluzzi L., Kepp O., Kroemer G. (2016). Mitochondrial Regulation of Cell Death: A Phylogenetically Conserved Control. Microb. Cell.

[62] Kessel D., Oleinick N.L. (2018). Cell Death Pathways Associated with Photodynamic Therapy: An Update. Photochem. Photobiol..

[63] Dos Santos A.F., Terra L.F., Wailemann R.A.M., Oliveira T.C., Gomes V.d.M., Mineiro M.F., Meotti F.C., Bruni-Cardoso A., Baptista M.S., Labriola L. (2017). Methylene Blue Photodynamic Therapy Induces Selective and Massive Cell Death in Human Breast Cancer Cells. BMC Cancer.

[64] Castano A.P., Demidova T.N., Hamblin M.R. (2005). Mechanisms in Photodynamic Therapy: Part Three—Photosensitizer Pharmacokinetics, Biodistribution, Tumor Localization and Modes of Tumor Destruction. Photodiagn. Photodyn. Ther..

[65] Menon M.B., Dhamija S. (2018). Beclin 1 Phosphorylation—At the Center of Autophagy Regulation. Front. Cell Dev. Biol..

[66] Wang W., Moriyama L.T., Bagnato V.S. (2012). Photodynamic Therapy Induced Vascular Damage: An Overview of Experimental PDT. Laser Phys. Lett..

[67] Van Vré Emily A., Ait-Oufella H., Tedgui A., Mallat Z. (2012). Apoptotic Cell Death and Efferocytosis in Atherosclerosis. Arterioscler. Thromb. Vasc. Biol..

[68] Zhang Y., Chen X., Gueydan C., Han J. (2018). Plasma Membrane Changes during Programmed Cell Deaths. Cell Res..

[69] Leibowitz B., Yu J. (2010). Mitochondrial Signaling in Cell Death via the Bcl-2 Family. Cancer Biol. Ther..

[70] Sun X., Zhang H., Zhang Y., Yang Q., Zhao S. (2018). Caspase-Dependent Mitochondrial Apoptotic Pathway Is Involved in Astilbin-Mediated Cytotoxicity in Breast Carcinoma Cells. Oncol. Rep..

[71] Wan Q., Liu L., Xing D., Chen Q. (2008). Bid Is Required in NPe6-PDT-Induced Apoptosis. Photochem. Photobiol..

[72] D’Arcy M.S. (2019). Cell Death: A Review of the Major Forms of Apoptosis, Necrosis and Autophagy. Cell Biol. Int..

[73] Reiners J.J., Agostinis P., Berg K., Oleinick N.L., Kessel D. (2010). Assessing Autophagy in the Context of Photodynamic Therapy. Autophagy.

[74] Garg A.D., Maes H., Romano E., Agostinis P. (2015). Autophagy, a Major Adaptation Pathway Shaping Cancer Cell Death and Anticancer Immunity Responses Following Photodynamic Therapy. Photochem. Photobiol. Sci..

[75] Allison R.R., Moghissi K. (2013). Photodynamic Therapy (PDT): PDT Mechanisms. Clin. Endosc..

[76] Garg A.D., Nowis D., Golab J., Agostinis P. (2010). Photodynamic Therapy: Illuminating the Road from Cell Death towards Anti-Tumour Immunity. Apoptosis.

[77] Cavaco A., Rezaei M., Niland S., Eble J.A. (2017). Collateral Damage Intended—Cancer-Associated Fibroblasts and Vasculature Are Potential Targets in Cancer Therapy. Int. J. Mol. Sci..

[78] Sia J., Szmyd R., Hau E., Gee H.E. (2020). Molecular Mechanisms of Radiation-Induced Cancer Cell Death: A Primer. Front. Cell Dev. Biol..

[79] Jarosz-Biej M., Smolarczyk R., Cichoń T., Kułach N. (2019). Tumor Microenvironment as A “Game Changer” in Cancer Radiotherapy. Int. J. Mol. Sci..

[80] Hassannia B., Vandenabeele P., Vanden Berghe T. (2019). Targeting Ferroptosis to Iron Out Cancer. Cancer Cell.

[81] Lu B., Chen X.B., Ying M.D., He Q.J., Cao J., Yang B. (2017). The Role of Ferroptosis in Cancer Development and Treatment Response. Front. Pharmacol..

[82] Khong H.T., Restifo N.P. (2002). Natural Selection of Tumor Variants in the Generation of “Tumor Escape” Phenotypes. Nat. Immunol..

[83] Gollnick S.O., Evans S.S., Baumann H., Owczarczak B., Maier P., Vaughan L., Wang W.C., Unger E., Henderson B.W. (2003). Role of Cytokines in Photodynamic Therapy-Induced Local and Systemic Inflammation. Br. J. Cancer.

[84] Evans S., Matthews W., Perry R., Fraker D., Norton J., Pass H.I. (1990). Effect of Photodynamic Therapy on Tumor Necrosis Factor Production by Murine Macrophages. J. Natl. Cancer Inst..

[85] Cecic I., Korbelik M. (2002). Mediators of Peripheral Blood Neutrophilia Induced by Photodynamic Therapy of Solid Tumors. Cancer Lett..

[86] Krysko D.V., Garg A.D., Kaczmarek A., Krysko O., Agostinis P., Vandenabeele P. (2012). Immunogenic Cell Death and DAMPs in Cancer Therapy. Nat. Rev. Cancer.

[87] Galluzzi L., Vitale I., Abrams J.M., Alnemri E.S., Baehrecke E.H., Blagosklonny M.V., Dawson T.M., Dawson V.L., El-Deiry W.S., Fulda S. (2012). Molecular Definitions of Cell Death Subroutines: Recommendations of the Nomenclature Committee on Cell Death 2012. Cell Death Differ..

